# Associations Between Psychological and Immunological Variables in Chronic Fatigue Syndrome/Myalgic Encephalomyelitis: A Systematic Review

**DOI:** 10.3389/fpsyt.2021.716320

**Published:** 2021-11-23

**Authors:** Emilie F. W. Raanes, Tore C. Stiles

**Affiliations:** Department of Psychology, Norwegian University of Science and Technology, Trondheim, Norway

**Keywords:** chronic fatigue syndrome, myalgic encephalomyelitis, executive function, emotion regulation, interpersonal function, sleep, immunological markers, psychoneuroimmunology

## Abstract

**Background:** Little emphasis has been given to the fact that various psychological processes and behaviors in chronic fatigue syndrome/myalgic encephalomyelitis (CFS/ME) have neural correlates that affect—and are affected by—the immune system. The aim of this paper is to provide a systematic review of the literature on cross-sectional and longitudinal associations between psychological and immunological variables/changes in CFS/ME.

**Methods:** The systematic literature search was conducted on Dec 10, 2020 using PubMed. Original research studies investigating associations between a predefined set of psychological and immunological variables in CFS/ME were included. Specifically, the review was focused on studies examining the following psychological variables: executive function, emotion regulation, interpersonal function, sleep, mental health, anxiety, depression, and/or other psychiatric symptoms. In terms of immunological variables, studies investigating interleukin (IL)-1, IL-2, IL-4, IL-6, tumor necrosis factor (TNF), CD4^+^, and/or CD8^+^ were included. Besides original research papers, other potentially relevant papers (e.g., literature reviews) were carefully read and reference lists were checked in order to identify any additional relevant studies. Available data was summarized in text and tables.

**Results:** The literature search identified 897 potentially relevant papers. Ultimately, 14 studies (807 participants in total) were included in the review of which only two were longitudinal in nature. The review indicated that executive function is associated with IL-1 and IL-6, and interpersonal function is associated with IL-6 and TNF-α. Further, the available data suggested that emotion regulation is associated with IL-2 and sleep is associated with IL-1, IL-6, TNF-α, and IL-2. Interestingly, poorer emotion regulation, interpersonal function, and sleep have all been found to be associated with higher cytokine levels. Executive function has shown both positive and negative relationships with cytokines and among these psychological constructs, it is also the only one that has been found to be associated with CD4^+^ and CD8^+^ counts/percentages.

**Conclusions:** Correlations exist between psychological and immunological variables in CFS/ME. However, there are few consistent findings and there is almost a complete lack of longitudinal studies. This review points to a gap in existing CFS/ME research and hopefully, it will inspire to the generation of innovative, psychoneuroimmunological hypotheses within the CFS/ME research field.

## Introduction

The main feature of chronic fatigue syndrome/myalgic encephalomyelitis (CFS/ME) is persistent physical and mental fatigue that is not alleviated by rest or sleep. However, the disease is heterogeneous and may present with a multitude of symptoms (1).

Patients with CFS/ME often report impaired attention, poor memory, and concentration difficulties (2). Confirming these subjective complaints, objective neuropsychological tests have demonstrated impaired executive functioning among patients, particularly in the domains of psychomotor speed and attention (3). Previous work has also confirmed the presence of objective impairments in working, visual, and verbal episodic memory (4). Some patients have an attentional bias to health-threatening and illness-related information (5).

Repetitive, negative thinking is common in CFS/ME and is often used as a means of regulating unpleasant emotions (6, 7). Relative to healthy control subjects, patients with CFS/ME further tend to suppress and avoid emotions (8, 9), which is another form of emotion regulation. Possibly, this could be due to patients' negative beliefs about the acceptability of experiencing and expressing negative thoughts and emotions (10). Research also suggests that CFS/ME patients have poorer emotional self-awareness (11), higher levels of alexithymia and poorer ability to recognize emotions in the faces of others (11, 12), compared with healthy controls. The cognitive and emotional difficulties experienced by CFS/ME patients may, in turn, have an adverse impact on social and occupational functioning.

The experience of unrefreshing or non-restorative sleep is very common in CFS/ME (13). However, objective measures of sleep architecture (polysomnography) have yielded inconsistent findings (14, 15). An alternative biological explanation has been posited, linking non-restorative-sleep to nocturnal autonomic nervous system (ANS) disturbance (16). Specifically, reduced parasympathetic activity during sleep has been suggested to represent a biological correlate of unrefreshing sleep (16–18).

A range of CFS/ME symptoms such as dizziness, nausea, cardiac arrhythmias, orthostatic intolerance, frequent urination, and abdominal discomfort, may be related to ANS dysfunction. Recent meta-analyses have revealed significant differences between CFS/ME patients and healthy controls in several heart rate parameters, collectively suggestive of reduced vagal (parasympathetic), and increased sympathetic modulation of heart rate (19, 20). Moreover, hypothalamic-pituitary-adrenal (HPA) axis changes as indicated by mild hypocorticolism, enhanced negative feedback to the HPA axis, attenuated diurnal variation of cortisol, and/or blunted HPA axis responsiveness to stress, have been demonstrated in many patients with CFS/ME (21, 22). Adding to the complexity of CFS/ME, it may also present with flu-like symptoms such as fever, headache, muscle and joint pain, sore throat, and swollen lymph nodes. Possibly, these physical symptoms could be related to the immunological alterations observed in CFS/ME patients (23, 24).

Psychology and immunology have traditionally been regarded as separate fields of study. However, psychoneuroimmunology emerged in the early 1980s and it is becoming increasingly clear that the nervous system affects—and is affected by—the immune system (25). This implies that neither of these systems operate autonomously but are interconnected; immunological processes can influence the brain and the mind, and the mind can play a role in health and disease. Underlying these interactions are bidirectional, humoral and neural communication pathways between the nervous system and the immune system (26–29). Two major pathways by which the nervous system affects the immune system are the ANS and the neuroendocrine system (e.g., the HPA axis).

Nearly one-third of patients with CFS/ME report substantial improvements in their health following psychological treatments (30, 31), suggesting that (parts of) the underlying pathogenic mechanism(s), perhaps in a subgroup of patients, can be modulated by favorable changes in psychological processes and/or health behaviors. Considering that CFS/ME is a multisystem disease, this notion gives rise to several unanswered questions that should be empirically examined. In patients that benefit from psychological treatments, are treatment-induced psychological and/or behavioral changes accompanied by similar changes in both fatigue and physiological parameters? In this context, do the ANS and the HPA axis act as mediators of change in immunological parameters? Which psychological and/or health behavior variables are most strongly associated with autonomic, endocrine, and immunological parameters? Would a psychological treatment targeting these particular variables be more effective in reducing level of fatigue? Providing answers to these questions will most likely improve the understanding of CFS/ME and may further be a crucial step toward therapeutic developments, which are urgently needed (32).

A thorough review of the literature on associations between psychological and immunological variables in CFS/ME has not been conducted since 2001 (33). The aim of this paper is thus to provide a systematic review of the literature on (i) cross-sectional associations between psychological and immunological variables, and (ii) longitudinal associations between changes in psychological variables and changes in immunological variables, in CFS/ME. Specifically, the present review is focused on studies investigating one or several of the key psychological variables that potentially can be targeted in a psychological treatment approach for CFS/ME, namely executive function, emotion regulation, interpersonal function, sleep, mental health, anxiety, depression, and other psychiatric symptoms. Among the many potentially relevant health behavior variables, we put special emphasis on sleep for several reasons; unrefreshing sleep is one of the hallmark symptoms of CFS/ME and sleep is a fundamental part of life, vital for mental and physical health (34, 35). The present review is further focused on studies investigating one or several of the following immunological variables: the cytokines interleukin (IL)-1, IL-2, IL-4, IL-6, and tumor necrosis factor (TNF) as well as T cells (CD4^+^ and CD8^+^ counts/percentages).

The present systematic review seeks to provide interested readers with an overview of the empirical state of the field concerning associations between specific psychological constructs (e.g., executive function) and specific immunological parameters.

## Methods

The systematic review was conducted according to the Preferred Reporting Items for Systematic reviews and Meta-Analyses (PRISMA) 2020 statement (36, 37). This systematic review was not registered, and a protocol was not prepared.

### Eligibility Criteria

Studies were assessed for inclusion in the review according to the following set of eligibility criteria: (i) study participants were adults (aged ≥18 years) diagnosed with CFS/ME, (ii) original research was reported, (iii) associations between psychological variables (executive function, emotion regulation, interpersonal function, sleep, mental health, anxiety, depression, and/or other psychiatric symptoms) and immunological variables (IL-1, IL-2, IL-4, IL-6, TNF, CD4^+^, and/or CD8^+^) were examined, (iv) the psychological variable(s) was assessed by either an independent, subjective measure (full scale or subscale) or an objective measure, and (v) the paper was available in English. Studies not meeting these criteria were excluded. Additionally, studies investigating various patient groups and CFS/ME case studies were excluded.

Any independent, subjective measure or objective measure of the eight psychological variables (executive function, emotion regulation, interpersonal function, sleep, mental health, anxiety, depression, and/or other psychiatric symptoms) was considered for inclusion in the review. In the context of cytokines, both direct and indirect measures (cytokine levels and cytokine receptor levels, respectively) were considered. With regard to analytical methods, the review was focused on correlation-, regression-, and SEM-based associations. Any criteria for CFS/ME diagnosis were accepted for a report to be considered for inclusion. As regards longitudinal studies, minimum time interval between measurements was not set. In the context of longitudinal treatment studies, both psychological and pharmacological treatment studies were considered. Full-text access was not a criterion for reports to be considered for inclusion as long as the essential data on associations between psychological and immunological variables was reported in the abstract and/or in a secondary reference (i.e., literature review). Strict inclusion criteria were not applied for the reason that little research has been conducted on the topic.

### Search Strategy

The systematic literature search was conducted on December 10, 2020 using PubMed. The database coverage was 1987 to present, and no filters were applied to limit the search results. Based on the eligibility criteria, however, search results could have been limited by language (English), species (humans), and age (adults). The following search query was used:

(((“chronic fatigue syndrome”) OR (“myalgic encephalomyelitis”)) AND ((“immune marker”) OR (“immune markers”) OR (“immunological marker”) OR (“immunological markers”) OR (“immune parameter”) OR (“immune parameters”) OR (“immunological parameter”) OR (“immunological parameters”) OR (“immunologic parameter”) OR (“immunologic parameters”) OR (“immune response”) OR (“immune responses”) OR (“white blood cell”) OR (“white blood cells”) OR (“T cell”) OR (“T cells”) OR (“B cell”) OR (“B cells”) OR (lymphocyte) OR (lymphocytes) OR (“natural killer cell”) OR (“natural killer cells”) OR (cytokine) OR (cytokines) OR (interleukin) OR (interleukins) OR (“tumor necrosis factor”) OR (inflammation))) AND ((“threat monitoring”) OR (“attentional bias”) OR (“threat bias”) OR (“cognitive bias”) OR (“symptom focusing”) OR (“executive function”) OR (“executive functioning”) OR (attention) OR (“attentional control”) OR (“psychomotor speed”) OR (alerting) OR (orienting) OR (“executive control”) OR (“cognitive control”) OR (“cognitive processing”) OR (memory) OR (“cognitive difficulties”) OR (“cognitive problems”) OR (“cognitive performance”) OR (“cognitive functioning”) OR (“emotion regulation”) OR (“affect regulation”) OR (“emotional regulation”) OR (“emotion”) OR (“stress management”) OR (“psychological stress”) OR (worry) OR (rumination) OR (“interpersonal behavior”) OR (behavior) OR (“coping behavior”) OR (“coping strategies”) OR (“social behavior”) OR (“interpersonal difficulties”) OR (“psychosocial functioning”) OR (“psychiatric symptoms”) OR (“symptom severity”) OR (“clinical parameters”) OR (“illness parameters”) OR (sleep) OR (pain) OR (fatigue))

When considered relevant to the topic of the present review, other article types besides original research papers (e.g., literature reviews) were carefully read in order to identify any additional relevant studies. In addition, the reference lists of papers that were not excluded during title and abstract screening were checked for any relevant studies not identified in the initial search.

### Search Strategy Development, Selection, and Data Collection Process

Keyword search terms were decided by both authors (ER and TS). Each author made a list of key concepts related to the research question, including synonyms and related words. In addition, the Ovid Search Builder was used to identify possible synonyms. Consensus on which search terms to include was reached by discussion.

The initial title and abstract screening were performed by one of the authors (ER). Careful reading of other potentially relevant papers besides original research papers was performed by the same author (ER). Both authors (ER and TS) independently assessed full-text papers for inclusion. As for reports that were not available in full text, the abstracts combined with the data available in the secondary references, were assessed for inclusion. In case of disagreement, consensus was reached on inclusion or exclusion by discussion. The reference lists of eligible papers were screened by one of the authors (ER).

Data from eligible studies was extracted and entered into tables (Microsoft Word) by one of the authors (ER). The following parameters were extracted: study authors, year of publication, study design, time interval between measurements (longitudinal studies only), country in which the study was conducted, sample size, mean age, percentage of females, criteria used for CFS/ME diagnosis, associations between psychological and immunological variables, and details on the measurement of psychological variables (self-report/objective measure). Unless otherwise specified, all data was extracted from the primary reference for each included study. When uncertainties arose with regard to interpretation of the data, the other author (TS) assisted in the data extraction process. Any disagreements were resolved by discussion.

### Synthesis Methods

Key characteristics of the included studies are presented in tables. In papers that provided information only on the number of male/female participants, percentage of female participants was calculated. Data on associations between psychological and immunological variables are mainly presented through text. Tables summarizing the results of individual studies were created to provide an overview of the data across various psychological and immunological variables. Both in text and in tables, findings are grouped based on the specific psychological or immunological variable investigated to facilitate identification of patterns in the data. Meta-analyses were not considered appropriate in the context of the present review due to several reasons (see Limitations section).

## Results

### Study Selection

**Figure 1:**
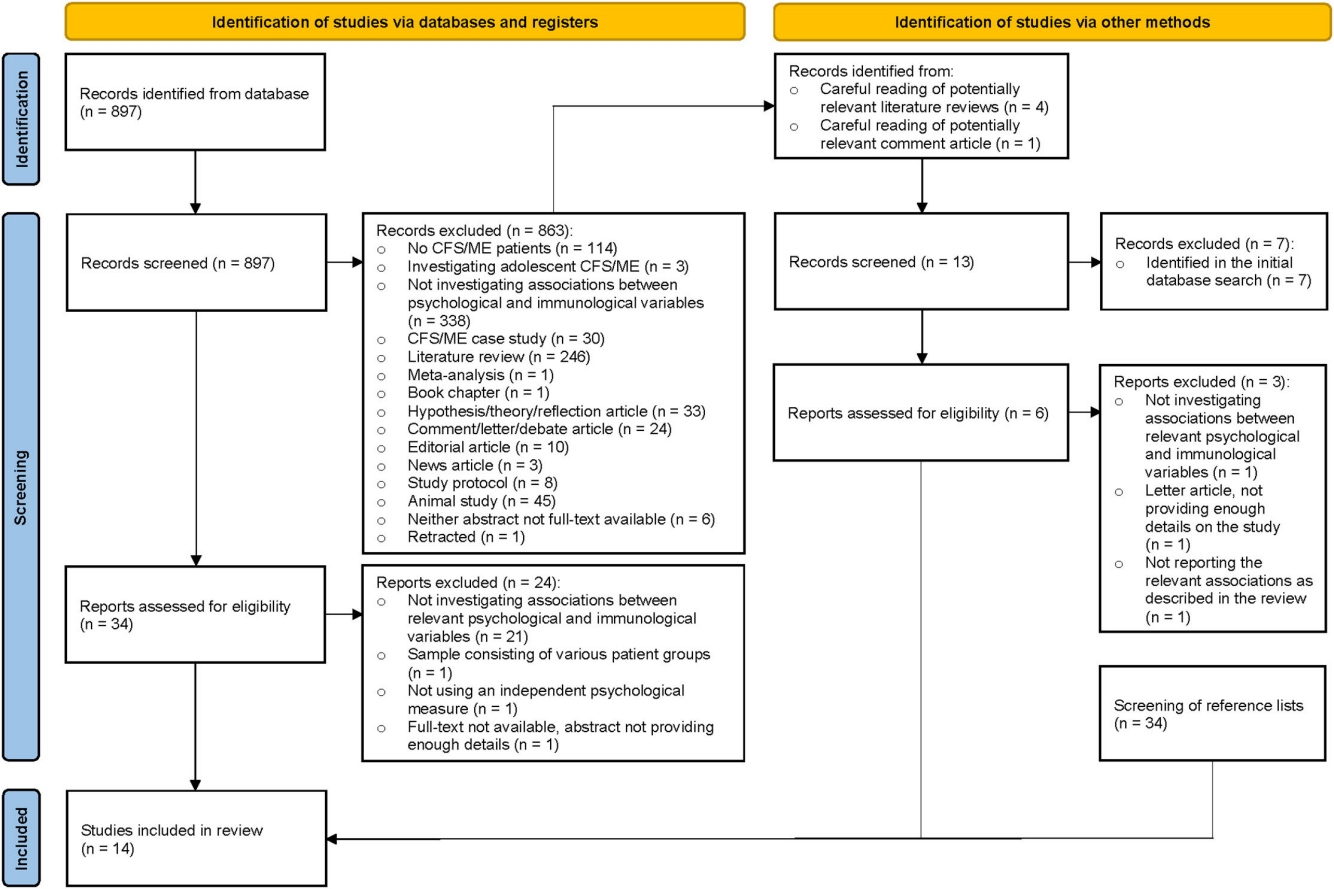
PRISMA flow-diagram summarizing the literature search and screening process (36).

The systematic literature search identified 897 potentially relevant records. During title and abstract screening, 863 records were excluded. An overview of reasons for exclusion is provided in the figure below. The remaining 34 full-text reports were assessed for eligibility of which 10 fulfilled the inclusion criteria (38–47). At this stage of the review process, reports were excluded based on the following reasons: not investigating/reporting associations between relevant psychological and immunological variables (*n* = 21) (24, 48–70); sample consisting of various patient groups (*n* = 1) (71); not using an independent psychological measure (*n* = 1) (72); full-text not available, abstract not providing enough details (*n* = 1) (73). Four of the records that were excluded during title and abstract screening were potentially relevant literature reviews (33, 74–76) and one of the excluded records was a potentially relevant comment article (77). From these papers, 13 potentially relevant records (24, 38, 39, 41, 42, 44, 67, 78–83) were identified. Seven of these records (24, 38, 39, 41, 42, 44, 67) were excluded during title and abstract screening as they had already been identified in the initial database search. The remaining six reports (78–83) were assessed for eligibility of which three (81–83) fulfilled the inclusion criteria (as for two of these reports, only abstracts were available). Reports were excluded based on the following reasons: not investigating associations between relevant psychological and immunological variables (*n* = 1) (78); letter article, not providing enough details on the study (*n* = 1) (79); not reporting the relevant associations as described in the review (n = 1) (80). Lastly, one report (only abstract available) (84) fulfilling the inclusion criteria was identified through screening of reference lists (*n* = 34). In total, 14 studies (807 participants in total) were included in the review (Figure 1).

### Study Characteristics

The studies included in the review were published between 1992 and 2018. Besides two studies, which were longitudinal in nature, the studies adopted either a case-control or cross-sectional design. One of the longitudinal studies was a randomized controlled trial testing the effect of a psychological treatment intervention for CFS/ME (47), and the other longitudinal study was a double blind, placebo-controlled trial testing the effect of high dose intravenous immunoglobulin (immunotherapy) for CFS/ME (40). Time interval between measurements were 4–6 and 5 months, respectively. Most of the included studies had a small sample size (ranging from 18 to 265 participants). Five reports did not provide information on mean age (as for three of these reports, only abstracts were available). Based on the available information, mean age ranged from 33.4 to 50.7 years of age. Four reports did not provide information on the percentage or number of female/male participants (as for three of these reports, only abstracts were available). However, females comprised the majority of the sample in most studies. Most of the included studies used the Centers for Disease Control and Prevention (CDC) 1994 (Fukuda) criteria for CFS/ME diagnosis (1). As for other diagnostic criteria, two studies used the Oxford 1991 (Sharpe) criteria (85), one study used the Australian 1990 (Lloyd) criteria (86) and two studies used the CDC 1988 (Holmes) criteria (87). Two reports did not provide information on the specific CFS/ME criteria used (only abstracts available). Citations and key characteristics of the included studies are displayed below (Table 1). Studies are listed by year of publication.

**Table 1:** Key characteristics of the included studies, including first author and year of publication, citation, study design, time interval between measurements (longitudinal studies only), country in which the study was conducted, sample size, mean age, percentage of females, and the criteria used for CFS/ME diagnosis.

**Study**				**Population**				
**First author (year)**	**Citation**	**Study design**	**Time interval between measurements**	**Country**	**Sample size**	**Age, mean**	**Female, %**	**CFS/ME criteria**
Groven (2018)^*^	(44)	Case-control	–	Norway	20	–	100	CDC 1994
Milrad (2018)	(42)	Cross-sectional	–	USA	265	49.4	81.7	CDC 1994
Milrad (2017)	(41)	Cross-sectional	–	USA	60	50.5	100	CDC 1994
Lattie (2012)	(39)	Cross-sectional	–	USA	117	50.7	82.9	CDC 1994
Nas (2011)	(45)	Case-control	–	Turkey	25	33.4	84	CDC 1994
Van Hoof (2007)	(43)	Cross-sectional	–	Brussels	48	45	60.4	CDC 1994
Kozora (2005)^**^	(84)	Case-control	–	USA	18	–	–	–
Cruess (2000)^**^	(82)	Cross-sectional	–	USA	27	–	–	–
Hassan (1998)	(46)	Case-control	–	UK	44	40.2	68.2	Oxford 1991
Borish (1998)	(38)	Case-control	–	USA	18	43.3	80	CDC 1988
Peakman (1997)	(47)	Longitudinal	4–6 months	UK	43	35	68.3	Oxford 1991
Lutgendorf (1995)^**^	(81)	Cross-sectional	–	USA	65	–	–	CDC 1994
Hickie (1992)	(40)	Longitudinal	5 months	Australia	33	36.5	45.5	Australian 1990
Klimas (1992)^*^	(83)	Cross-sectional	–	USA	24	–	–	CDC 1988

* *The report did not provide information on mean age/percentage of females*;

** *the full-text paper was not available, and the abstract/secondary reference did not provide information on mean age/percentage of females/diagnostic criteria; CDC, Centers for Disease Control and Prevention*.

The present review was structured around 10 categories of psychological variables: executive function, emotion regulation, interpersonal function, sleep, mental health, anxiety, depression, other psychiatric symptoms, fatigue, and pain. Details on measurement (self-report/objective measures), the number of studies investigating variables related to each category and the associated citations are presented above (Table 2). Importantly, the current paper did not aim to provide a systematic review of the literature on associations between fatigue/pain and immunological parameters in CFS/ME and only provides an overview of such associations in the context of the studies included in the review.

**Table 2:** Categories of psychological variables, details on measurement, the number of studies investigating variables related to each category, and the associated citations.

**Psychological variable category**	**Measurement**	**No. of studies**	**Citation(s)**
Executive function	Self-report	3	(81–83)
	Neuropsychological test	2	(83, 84)
Emotion regulation	Self-report	2	(39, 45)
Interpersonal function	Self-report	3	(44–46)
Sleep	Self-report	3	(41, 45, 82)
	Polysomnography	1	(43)
Mental health	Self-report	2	(46, 47)
Anxiety	Self-report	1	(44)
Depression	Self-report	5	(40, 42, 44, 45, 47)
Other psychiatric symptoms	Self-report	3	(38, 44, 83)
Fatigue^*^	Self-report	7	(39, 41, 44–47, 83)
Pain^*^	Self-report	2	(45, 46)

* *The current paper did not aim to provide a systematic review of the literature on associations between fatigue/pain and immunological variables in CFS/ME and only provides an overview of such associations in the context of the studies included in the review*.

### Cross-Sectional Associations

The cross-sectional associations (correlations, unless otherwise stated) between psychological and immunological variables in CFS/ME, including the baseline associations reported in the two longitudinal studies, are presented in the following sections.

#### Executive Function

No studies have yet investigated the associations between subjective executive function and IL-1, IL-2, IL-4, IL-6, or TNF. However, one study has demonstrated significant associations between objective executive function and cytokines, including IL-1β and IL-6 (84). Specifically, better performance on the Brief Visuospatial Memory Test-Learning (better visuospatial learning) and lower scores on Letter Fluency (poorer lexical access ability and executive control ability) were found to be associated with higher levels of IL-1β, and better performance on the Trail Making Test A and B (better visual attention, processing speed, set shifting, and mental flexibility) were found to be associated with higher levels of IL-6 (84). No studies have investigated the associations between objective executive function skills and IL-2, IL-4, or TNF. Further, two studies have shown significant associations between subjective executive function and T cells (33, 81, 82). In one of these studies, higher levels of cognitive difficulties were found to be associated with higher CD4^+^ and lower CD8^+^ counts (81). In the other study, higher levels of cognitive difficulties were found to be associated with a lower CD8^+^ percentage (only abstract available; details were extracted from a literature review) (33, 82). Yet another study has demonstrated a significant association between objective executive function and T cells (83). Specifically, poorer visual reproduction was found to be associated with a lower CD4^+^ percentage (83).

#### Emotion Regulation

One study has shown a significant association between emotion regulation (stress management skills) and IL-2 (39). Specifically, lower perceived ability to enact stress management skills were found to be associated with higher levels of IL-2. However, the same study also showed no significant association between emotional distress and IL-2 (39). Further, in another study, no significant association was found between emotion regulation (emotional reactions) and IL-2 receptor (IL-2R) (45). Significant associations between emotion regulation (stress management skills, emotional reactions) and the cytokines IL-1β, IL-6, and TNF-α have not been demonstrated (39, 45), and the association between emotion regulation and IL-4 has not been investigated to date. Further, significant associations between emotion regulation (emotional reactions) and T cells (CD4^+^ and CD8^+^ counts) have not been found (45).

#### Interpersonal Function

One study has demonstrated a significant association between interpersonal function and IL-6 (45). Specifically, higher levels of social isolation were found to be associated with higher levels of IL-6 (45). In another study, no significant association was found between interpersonal function (interpersonal sensitivity) and IL-6 (44). Further, no significant associations have been found between interpersonal function (social isolation, interpersonal sensitivity) and the cytokines IL-2R, IL-4, and TNF-α (44, 45). No study has yet examined the association between interpersonal function and IL-1. Neither have any significant associations between interpersonal function (social isolation, social functioning) and T cells (CD4^+^ and CD8^+^ counts/percentages) been demonstrated (45, 46).

#### Sleep

Two studies have demonstrated significant associations between subjective sleep and cytokines, including IL-1β, IL-6, and TNF-α (41, 45). In one of these studies, poorer sleep quality and quantity were found to be associated with higher levels of IL-1β, IL-6, and TNF-α (regression analysis) (41). Likewise, the other study found that higher levels of sleeplessness were associated with higher levels of IL-6 (45). In the latter study, higher levels of sleeplessness were also found to be associated with higher levels of IL-2R (45). No study has examined the association between subjective sleep and IL-4. Neither has any study investigated the associations between objective sleep and IL-1, IL-2, IL-4, IL-6, or TNF. Further, no significant associations have been found between subjective sleep (sleeplessness) and T cells (CD4^+^ and CD8^+^ counts) (45). Neither has any significant association between objective (alpha-delta) sleep and CD8^+^ cells (count) been demonstrated (43), and the associations between objective sleep parameters and CD4^+^ cells have not been investigated to date.

#### Mental Health

No previous study has examined the associations between mental health and IL-1, IL-2, IL-4, IL-6, or TNF. However, one study has demonstrated several significant associations between mental health and T cells (46). Specifically, worse mental health was found to be associated with a lower CD4^+^ count, a lower CD4^+^ percentage and a higher CD8^+^ percentage. Similarly, higher role limitations due to emotional problems were found to be associated with a lower CD4^+^ count and a higher CD8^+^ percentage (46). However, no significant associations between mental health/role limitations due to emotional problems and T cells (CD4^+^ count/percentage and CD8^+^ percentage) have also been demonstrated (46, 47).

#### Anxiety

No significant associations have been found between anxiety/phobic anxiety and the cytokines IL-4, IL-6, and TNF-α (44). Further, no previous study has examined the associations between anxiety and IL-1, IL-2, or CD4^+^/CD8^+^ T cells.

#### Depression

One study has demonstrated a significant association between depression and IL-6 (45). Specifically, higher levels of depression were found to be associated with higher levels of IL-6 (45). In another study, no significant association was found between depression and IL-6 (44). Further, significant associations between depression and the cytokines IL-2R, IL-4, and TNF-α have not been found (44, 45). Interestingly, however, one study has demonstrated a significant association between higher levels of depressive symptoms and higher levels of inflammation, collectively indicated by higher IL-2, IL-6, and TNF-α levels (SEM analysis) (42). No previous study has examined the association between depression and IL-1, and no significant associations have been demonstrated between depressive symptoms and T cells (CD4^+^ and CD8^+^ counts/percentages) (40, 45, 47).

#### Other Psychiatric Symptoms

One study has demonstrated significant associations between other psychiatric symptoms and TNF-α (regression analysis) (38). First, the presence of a personality disorder was found to be associated with lower levels of TNF-α. Second, higher global psychiatric symptom intensity was found to be associated with higher levels of TNF-α (38). In another study, no significant associations were found between other psychiatric symptoms, including obsessive compulsion, paranoid ideation, psychoticism, hostility, and somatization, and the cytokines IL-4, IL-6, and TNF-α (44). No previous study has examined the associations between other psychiatric symptoms and the cytokines IL-1 and IL-2. Furthermore, one study has demonstrated significant associations between other psychiatric symptoms and T cells (83). Specifically, more anti-social, sadistic, and passive-aggressive personality styles as well as higher levels of alcohol and drug dependence were found to be associated with higher CD4^+^ counts (83). No study has examined the associations between other psychiatric symptoms and CD8^+^ cells.

#### Fatigue

Among the studies included in the current review, none have demonstrated significant associations between fatigue/energy level and cytokines, including IL-1β, IL-2(R), IL-4, IL-6, and TNF-α (39, 41, 44, 45). Further, one study has demonstrated significant associations between fatigue and T cells (47). Specifically, higher levels of fatigue were found to be associated with a higher CD4^+^ percentage and a lower CD8^+^ percentage (47). No significant associations between fatigue/energy level/energy/vitality and T cells (CD4^+^ and CD8^+^ counts/percentages) have also been demonstrated (45, 46). Importantly, this paper did not aim to provide a systematic review of the literature on associations between fatigue and immunological variables in CFS/ME.

#### Pain

Among the studies included in the present review, no significant associations have been found between pain and the cytokines IL-2R and IL-6 (45). The associations between pain and IL-1, IL-4, or TNF have not been investigated. Further, no significant associations have been found between (bodily) pain and T cells (CD4^+^ and CD8^+^ counts/percentages) (45, 46). As previously noted, this paper did not aim to provide a systematic review of the literature on associations between pain and immunological variables in CFS/ME.

### Longitudinal Associations

The longitudinal associations (correlations) between changes in psychological variables and changes in immunological variables are presented in the following section.

#### Depression

A pharmacological treatment study has demonstrated a significant association between changes (percentage decrease) in depressive symptoms and changes (percentage increase) in CD4^+^ cells (count) following intravenous immunoglobulin-based immunotherapy (40). No significant association was found between changes in depressive symptoms and changes in CD8^+^ cells (count) following immunotherapy. Similarly, no significant associations were found between changes in depressive symptoms and changes in T cells (CD4^+^ and CD8^+^ counts) following placebo therapy (40).

## Discussion

### Cross-Sectional Associations

#### Associations Between Psychological Variables and Cytokines

Poorer objective executive function skills (various) have been found to be associated with higher and lower levels of IL-1β as well as lower levels of IL-6 (84). These positive and negative associations are suggestive of intricate relationships between various aspects of executive function, as measured objectively by neuropsychological tests, and the cytokines IL-1 and IL-6 in CFS/ME.

Poorer emotion regulation (lower ability to enact stress management skills) has been found to be significantly associated with higher levels of IL-2 (39). No significant associations between emotion regulation (stress management skills, emotional distress, and emotional reactions) and cytokines, including IL-1β, IL-2(R), IL-6, and TNF-α, have also been demonstrated (39, 45). These latter findings are somewhat contrary to expectations considering the tight link between emotions and peripheral physiological responses (activation of the sympathetic division of the ANS and the HPA axis). Available data thus seems to suggest that some aspects of emotion regulation are related to IL-2.

Poorer interpersonal function (higher levels of social isolation) has been found to be significantly associated with higher levels of IL-6 (45). No significant associations between interpersonal function (social isolation, interpersonal sensitivity) and cytokines, including IL-2R, IL-4, IL-6, and TNF-α, have also been found (44, 45). On this basis, existing evidence indicates that some aspects of interpersonal behavior are linked to IL-6 in CFS/ME.

Poorer subjective sleep (poorer sleep quality and quantity) has been found to be significantly associated with higher levels of IL-1β, IL-2R, IL-6, and TNF-α (41, 45). Thus, sleep differs from the other psychological constructs in that sleep, as measured subjectively, seems to be associated with both IL-1β, IL-2R, IL-6, and TNF-α.

No significant associations have been found between (phobic) anxiety and cytokines, including IL-4, IL-6, and TNF-α (44). Thus, there is no evidence to suggest any relationships between anxiety and these cytokines in CFS/ME.

Higher levels of depressive symptoms have been found to be associated with higher levels of IL-6 (45). Higher levels of depressive symptoms have also been found to be associated with higher levels of inflammation as indicated by higher IL-2, IL-6, and TNF-α levels (SEM analysis) (42). No significant associations between depression and cytokines, including IL-2R, IL-4, IL-6, and TNF-α, have also been demonstrated (44, 45). These findings, however, may suggest that depressive symptoms are linked to both IL-2, IL-6, and TNF-α.

No significant associations have been found between other psychiatric symptoms, including obsessive compulsion, paranoid ideation, psychoticism, hostility and somatization, and the cytokines IL-4, IL-6, and TNF-α (44). The presence of a personality disorder has, however, been found to be associated with lower levels of TNF-α (38). In addition, higher global psychiatric symptom intensity (depression, anxiety, phobic anxiety, obsessive compulsion, paranoid ideation, psychoticism, hostility, and somatization) has been found to be associated with higher levels of TNF-α (38). As such, available data indicates that the presence of a personality disorder and possibly other types of psychiatric symptoms may be associated with TNF-α.

Among the studies included in this review, no significant associations have been found between fatigue/energy level and cytokines, including IL-1β, IL-2(R), IL-4, IL-6, and TNF-α (39, 41, 44, 45). Likewise, no significant associations have been found between pain and the cytokines IL-2R and IL-6 (45). Although fatigue- and pain-related findings must be interpreted with utmost caution, these findings might indicate that neither IL-1β, IL-2(R), IL-4, IL-6, or TNF-α are closely associated with fatigue in CFS/ME. Further, there is no evidence to suggest any relationships between pain and the cytokines IL-2R and IL-6.

#### Associations Between Psychological Variables and T Cells

Poorer subjective executive function (higher levels of cognitive difficulties) has been found to be significantly associated with a higher CD4^+^ count and a lower CD8^+^ count/percentage (33, 81, 82). In addition, poorer objective executive function (poorer visual reproduction) has been found to be significantly associated with a lower CD4^+^ percentage (83). Accordingly, both positive and negative relationships may exist between various subjective and objective executive function skills and CD4^+^/CD8^+^ T cells in CFS/ME.

Worse mental health/higher role limitations due to emotional problems has been found to be significantly associated with a lower CD4^+^ count/percentage and a higher CD8^+^ percentage (46). However, no significant associations between mental health/role limitations due to emotional problems and T cells (CD4^+^ count/percentage and CD8^+^ percentage) have also been demonstrated (46, 47). Available data might thus suggest that some aspects of mental health are related to CD4^+^/CD8^+^ T cells.

No significant associations between depressive symptoms and T cells (CD4^+^ and CD8^+^ counts/percentages) have been demonstrated (40, 45, 47). Accordingly, there is no evidence to suggest a relationship between depression and CD4^+^/CD8^+^ T cells in CFS/ME.

More anti-social, sadistic and passive-aggressive personality styles as well as higher levels of alcohol and drug dependence have been found to be significantly associated with higher CD4^+^ counts (83). Interestingly, these findings suggest a connection between certain personality traits and CD4^+^ T cells.

Among the studies included in this review, higher levels of fatigue have been found to be significantly associated with a higher CD4^+^ and a lower CD8^+^ percentage (47). Accordingly, the main symptom of CFS/ME, namely fatigue, might be associated with T cells. However, no significant associations between fatigue (energy level, energy/vitality) and T cells (CD4^+^ and CD8^+^ counts/percentages) have also been demonstrated (45, 46).

None of the remaining variables, including emotion regulation (emotional reactions), interpersonal function (social isolation, social functioning), subjective sleep (sleeplessness), objective (alpha-delta) sleep and (bodily) pain, have been found to be significantly associated with T cells (CD4^+^ and/or CD8^+^ counts/percentages) (43, 45, 46). An overview of the directions of the significant, cross-sectional associations between psychological and immunological variables in CFS/ME is presented above (Table 3). Replicated findings are marked in bold.

**Table 3:** The directions of the significant, cross-sectional associations between psychological and immunological variables in CFS/ME.

**Psychological variable category**	**Immunological variable**	**Citation(s)**
**Poorer executive function**		
Objective	↑IL-1β	(84)
Objective	↓IL-1β	(84)
Objective	↓IL-6	(84)
Subjective	↑CD4^+^ count	(81)
Objective	↓CD4^+^ %	(83)
Subjective	↓CD8^+^ count	(81)
Subjective	↓CD8^+^ %	(82) in (33)^*^
**Poorer emotion regulation**		
Subjective	↑IL-2	(39)
**Poorer interpersonal function**		
Subjective	↑IL-6	(45)
**Poorer sleep**		
Subjective	↑IL-1β	(41)
Subjective	**↑IL-6**	(41, 45)
Subjective	↑TNF-α	(41)
Subjective	↑IL-2R	(45)
**Poorer mental health**		
Subjective	↓CD4^+^ count	(46)
Subjective	↓CD4^+^ %	(46)
Subjective	↑CD8^+^ %	(46)
**Higher levels of depressive symptoms**		
Subjective	↑IL-2	(42)
Subjective	**↑IL-6**	(42, 45)
Subjective	↑TNF-α	(42)
**The presence of a personality disorder**		
Subjective	↓TNF-α	(38)
**More anti-social, sadistic, and passive-aggressive personality styles**		
Subjective	↑CD4^+^ count	(83)
**Higher levels of alcohol and drug dependence**		
Subjective	↑CD4^+^ count	(83)
**Higher global psychiatric symptom intensity**		
Subjective	↑TNF-α	(38)
Higher levels of fatigue^**^		
Subjective	↑CD4^+^ %	(47)
Subjective	↓CD8^+^ %	(47)

*↓, lower levels of (immunological marker); ↑, higher levels of (immunological marker); replicated findings are marked in bold*;

* *details on the association were extracted from a literature review (33)*;

** *the current paper did not aim to provide a systematic review of the literature on associations between fatigue and immunological variables in CFS/ME and only provides an overview of such associations in the context of the studies included in the review; all studies used correlation analyses besides two studies (38, 41) which used regression analyses and one study (42) which used SEM analysis*.

#### The Current Evidence Base on Cross-Sectional Associations

Although the only replicated findings are the associations between subjective sleep and IL-6 (41, 45) and between depression and IL-6 (42, 45), available cross-sectional data indicates that many key psychological constructs (executive function, emotion regulation, interpersonal function, sleep, and psychiatric symptoms) might be associated with cytokines, including IL-1β, IL-2(R), IL-6, and TNF-α (38, 39, 41, 42, 45, 84). Interesting in this context, poorer executive function, emotion regulation, interpersonal function and sleep as well as higher levels of depressive symptoms and higher global psychiatric symptom intensity, have all been found to be associated with higher cytokine levels (38, 39, 41, 42, 45, 84). However, various executive function skills have shown both positive and negative relationships with cytokines (84). Further, no study has demonstrated significant associations between psychological variables and IL-4. Based on the studies included in the present review, core CFS/ME psychophysical symptoms such as fatigue and pain do not seem to be associated with cytokines (39, 41, 44, 45).

Evidence further indicates that various symptom dimensions (mental health, psychiatric symptoms, and fatigue) might be associated with T cells (CD4^+^ and/or CD8^+^ counts/percentages) (33, 46, 47, 83), while emotion regulation, interpersonal function, and sleep are not (43, 45, 46). Among the psychological constructs examined in this review, only executive function has been found to be associated with T cells (CD4^+^ and CD8^+^ counts/percentages) (33, 81–83). Interestingly, poorer subjective executive function, more anti-social, sadistic, and passive-aggressive personality styles as well as higher levels of alcohol and drug dependence have all been found to be associated with higher CD4^+^ counts (81, 83). Further, poorer objective executive function and poorer mental health have both been found to be associated with lower CD4^+^ percentages (46, 83). Additionally, poorer subjective executive function and higher levels of fatigue have been found to be associated with lower CD8^+^ percentages (33, 47, 82). Based on the studies included in the review, pain does not seem to be associated with T cells (CD4^+^ and CD8^+^ counts/percentages) (45, 46). An overview of the current evidence base on cross-sectional associations between psychological and immunological variables in CFS/ME is presented below (Table 4). Specifically, Table 4 summarizes the number of studies showing a significant association between a psychological construct and an immunological variable relative to the number of studies that have examined this association. The associated citations are presented in brackets.

**Table 4:** An overview of the current evidence base on cross-sectional associations between psychological and immunological variables in CFS/ME.

**Psychological variable category**	**IL-1**	**IL-2(R)**	**IL-4**	**IL-6**	**TNF**	**CD4^**+**^**	**CD8^**+**^**
**Executive function**							
Subjective	NA	NA	NA	NA	NA	1/1 (81)	2/2 (81, 82)
Objective	1/1 (84)	NA	NA	1/1 (84)	NA	1/1 (83)	NA
**Emotion regulation**							
Subjective	0/1 (39)	1/2 (39, 45)	NA	0/2 (39, 45)	0/1 (39)	0/1 (45)	0/1 (45)
**Interpersonal function**							
Subjective	NA	0/1 (45)	0/1 (44)	1/2 (44, 45)	0/1 (44)	0/2 (45, 46)	0/2 (45, 46)
**Sleep**							
Subjective	1/1 (41)	1/1 (45)	NA	2/2 (41, 45)	1/1 (41)	0/1 (45)	0/1 (45)
Objective	NA	NA	NA	NA	NA	NA	0/1 (43)
**Mental health**							
Subjective	NA	NA	NA	NA	NA	1/2 (46, 47)	1/2 (46, 47)
**Anxiety**							
Subjective	NA	NA	0/1 (44)	0/1 (44)	0/1 (44)	NA	NA
**Depression**							
Subjective	NA	1/2 (42, 45)	0/1 (44)	2/3 (42, 44, 45)	1/2 (42, 44)	0/3 (40, 45, 47)	0/3 (40, 45, 47)
**Other psychiatric symptoms**							
Subjective	NA	NA	0/1 (44)	0/1 (44)	1/2 (38, 44)	1/1 (83)	NA
Fatigue^**^							
Subjective	0/2 (39, 41)	0/2 (39, 45)	0/1 (44)	0/4 (39, 41, 44, 45)	0/3 (39, 41, 44)	1/3 (45–47)	1/3 (45–47)
Pain^**^							
Subjective	NA	0/1 (45)	NA	0/1 (45)	NA	0/2 (45, 46)	0/2 (45, 46)

*This table summarizes the number of studies showing a significant association between a psychological construct and an immunological variable relative to the number of studies that have examined this association; the associated citations are presented in brackets; NA, not assessed. All studies used correlation analyses besides two studies (38, 41) which used regression analyses and one study (42) which used SEM analysis*;

** *the current paper did not aim to provide a systematic review of the literature on associations between fatigue/pain and immunological variables in CFS/ME and only provides an overview of such associations in the context of the studies included in the review*.

### Longitudinal Associations

Two of the studies included in this review adopted a longitudinal design. One of these studies was a pharmacological treatment study and the other was a psychological treatment study. In the pharmacological treatment study, percentage decrease in depressive symptoms was found to be significantly associated with percentage increase in CD4^+^ count following intravenous immunoglobulin-based immunotherapy (40). Although the associations between depressive symptom changes and other immunological changes did not reach a statistically significant level, patients receiving immunotherapy demonstrated a consistent pattern of positive associations between percentage decrease in depressive symptoms and percentage increase or improvement in markers of cell-mediated immunity. In the placebo therapy group, there was no such pattern of positive associations between psychological and immunological changes (40). These findings suggest that depression and immunological dysfunction in CFS/ME share a common pathophysiological mechanism. Alternatively, depression might occur secondary to immunological dysfunction (40). However, these results do not exclude the possibility that a psychological treatment may have favorable effects on both depression and immune system function in CFS/ME.

The psychological treatment study confirmed the presence of abnormal distributions of lymphocyte subsets in patients with CFS/ME (47). Despite clinical improvement over time, cognitive behavior therapy had no detectable impact on immunological parameters, including CD4^+^/CD8^+^ counts. Equally, immune status did not predict response or lack of response to treatment (47). These findings do not support the hypothesis that psychotherapy-induced psychological and behavioral changes are accompanied by changes in both fatigue and immunological parameters. Rather, these findings suggest that symptom severity is unrelated to immunological status in CFS/ME. However, it is also possible that psychotherapy-induced immunological changes are better reflected by measuring other immunological variables besides lymphocyte subsets (e.g., specific cytokines). Further, this study did not examine longitudinal associations between psychological and immunological changes following treatment completion. Although no immunological variable changed significantly over time, it cannot be excluded that psychological changes were significantly associated with immunological changes. Based on the results of this study, however, there is yet no evidence to suggest that changes in psychological variables during a psychological treatment for CFS/ME result in similar changes in immunological variables.

### Hypotheses and Recommendations for Future Research

Few studies have examined the associations between psychological and immunological variables in CFS/ME and the results of the present systematic review must, therefore, be treated with caution. However, we are hopeful that the present review will prove to be a useful, up-to-date tool for researchers seeking to further explore the psychoneuroimmunology of CFS/ME. The hypotheses in the following sections are based on the assumptions that immune dysfunction is a maintaining factor in CFS/ME and that psychological interventions may improve immune system function. According to a recent systematic review and meta-analysis including 56 studies on patients with various psychiatric disorders and physical diseases, psychosocial interventions can be reliably associated with enhanced immune system function (88). Psychological interventions may thus represent a viable strategy for reducing disease burden and improving health.

A psychological treatment approach for CFS/ME could potentially target the psychological processes and/or behavioral patterns underlying executive function, emotion regulation, interpersonal function, and sleep. As previously mentioned, poorer emotion regulation, interpersonal function, and sleep have all been found to be associated with higher cytokine levels (39, 41, 45). Although various executive function skills have shown both positive and negative relationships with cytokines, it can be hypothesized that psychotherapy-induced improvements in some aspects of executive function, emotion regulation, interpersonal function, and sleep would result in decreased cytokine production. More specifically, it can be hypothesized that (i) improvements in some aspects of executive and interpersonal functioning as well as sleep will contribute to reduce the levels of IL-1, IL-6, and TNF, and (ii) improvements in some aspects of emotion regulation and sleep will contribute to reduce the levels of IL-2. As executive function also has been found to be associated with T cells (CD4^+^ and CD8^+^ counts/percentages) (33, 81–83), it can further be hypothesized that improvements in some aspects of executive function would result in favorable changes in T cell counts/percentages. A normalization of immune system function in CFS/ME could, in turn, have reciprocal effects on the brain processes underlying executive function, emotion regulation, interpersonal function, and sleep, such creating a positive self-reinforcing mechanism facilitating recovery. Indeed, evidence indicates that circulating cytokines may cross the blood-brain barrier in sufficient amounts to affect brain function (89, 90).

Despite being two different conditions, sickness behavior and CFS/ME show a phenomenological overlap, both presenting with psychological and behavioral symptoms such as fatigue, malaise, hyperalgesia, sleepiness, neurocognitive symptoms, mood symptoms and possibly, post-exertional malaise (91). Sickness behavior is mainly induced by pro-inflammatory cytokines, particularly IL-1β, IL-6, and TNF-α, acting on the brain (92). Thus, it is likely that lower levels of IL-1, IL-6, and/or TNF following treatment would contribute to reduce sickness behavior among patients. As such, elevated levels of these cytokines might be a main factor underlying the fatigue experience. However, complex immunological reactions involving other immunological markers might contribute to drive the chronicity of CFS/ME. The degree to which reductions in cytokine levels and/or improvements in markers of immunity contribute to reduced symptom burden and recovery from CFS/ME is an important avenue for future research.

An important question for future research is whether improvements in immune system function in fact contribute to lower levels of fatigue. Based on the studies included in this review, it seems like the pathophysiological processes underlying fatigue are more directly linked with, or better reflected by, the alterations in T cells (CD4^+^ and CD8^+^ counts/percentages) than the cytokine alterations (IL-1, IL-2, IL-4, IL-6, and TNF) observed in CFS/ME. As previously mentioned, cytokines may, however, induce subjective feelings of sickness in the form of fatigue and other symptoms. Further, cytokine and T cell activity are connected. Immune cells, including T cells, act by releasing cytokines and cytokines are important to many aspects of T cell function (e.g., differentiation, activation, and proliferation) (93). Additionally, the current paper did not aim to provide a systematic review of the literature on associations between fatigue and immunological markers in CFS/ME. As yet, it is unknown whether improvements in immune system function contribute to lower levels of fatigue in CFS/ME. This central question sets the stage for further investigation.

On the one hand, the CFS/ME condition might reflect an immunological disturbance induced by the key biological systems that respond to stress (the ANS and the HPA axis). Possibly, this stress-related immunological disturbance could, in part, be maintained by psychological and health-related factors such as poor executive functioning and quality of sleep. Further, this stress-related immunological disturbance could possibly be reversed, at least to some degree, by favorable changes in psychological processes and health behaviors. On the other hand, CFS/ME might reflect a primary dysfunction within the immune system (e.g., autoimmune response). Chronic fatigue and an array of additional symptoms may thus develop as a product of dysregulated multisystemic interactions. In this case, CFS/ME would be classified as an immunological disease not related to psychological processes and health behaviors *per se*, and the underlying disease mechanism(s) would, therefore, not respond to favorable psychological/behavioral changes. However, it is more likely that several CFS/ME subgroups exist, each characterized by a specific kind of immunological disturbance. The bottom line is that a subgroup of CFS/ME patients may benefit from psychological treatments while another subgroup may need an alternative/pharmacological treatment intervention. Such subgroup classifications would also contribute to explain why some patients benefit from psychological interventions while some do not. CFS/ME subgroups may further be identified based on patients' immunological profiles. Exploring such subgroup classifications may pave the way for personalized CFS/ME treatment and is, therefore, a key research area for the future.

Assuming that CFS/ME symptoms are maintained by a disturbance in a specific part of the immune system, it must, from a psychoneuroimmunological point of view, be demonstrated that a psychological treatment can affect this part of the immune system in such a way that the immunological disturbance is ameliorated or eliminated. We suggest that future randomized controlled trials seek to provide empirical evidence that a specific psychological treatment facilitates improvements in specific psychological variables, and that these improvements are significantly associated with pre-post-changes in specific immunological parameters. Based on the findings of the present review, the quality of sleep might be an effective target of intervention. However, various psychological variables (e.g., executive, emotion regulation, interpersonal function, and psychiatric symptoms) should be explored as targets of intervention. Given that immunological alterations are a key maintaining factor in CFS/ME, the psychological treatment for CFS/ME will remain speculative until longitudinal associations between psychological and immunological changes have been demonstrated. In the context of a psychological treatment process, it is also essential that future studies seek to uncover the neuro-immune pathways by which psychological/behavioral changes affect the immune system. For example, the neural bases of emotions and emotion regulation involve the activation of limbic structures and a set of cortical regions (94), and improvements in emotion regulation during treatment would thus be accompanied by changes in neural activity in these regions. Through which neuro-immune pathways of communication are these changes in neural activity affecting the immune system? Based on previous work within the field of psychoneuroimmunology, it can be hypothesized that the ANS and the HPA axis are key mediators of change.

### Limitations

The present review is subject to some limitations that should be taken into consideration. PubMed was the only database used to identify eligible studies. A second literature search was, however, conducted using EMBASE and no eligible study was identified that was not already identified using PubMed. Few papers met the eligibility criteria and for simplification purposes, only the literature search and screening process in PubMed were reported. Further, eligibility was restricted to studies in English only. However, the systematic literature search in PubMed was not restricted by language and only one potentially relevant literature review was identified that was not available in English. Although the essential data on associations between psychological and immunological variables was available for all the included studies, we were unable to access the full text of three papers. Neither did we attempt to gain access to the full text of these papers by contacting study authors. Title and abstract screening, careful reading of other potentially relevant papers besides original research papers, screening of reference lists and data extraction were performed by only one of the review authors. However, both authors independently assessed full-text papers (when available) for inclusion. Furthermore, risk of bias in the included studies, risk of bias due to missing results, and certainty in the body of evidence were not thoroughly assessed. Based on the limited evidence base, we did not consider such assessments necessary at this point.

The studies included in the present systematic review used various criteria for diagnosing CFS/ME, different psychological measures, and varied assay systems to measure cytokines/T cells. Due to the limited number of published studies, it was, however, not appropriate to examine differences in study results according to various diagnostic criteria, psychological measures, and assay systems. Few studies were eligible for inclusion in the review and most of the included studies had a small number of participants, raising concerns about the accuracy and precision of measurements. The findings of the review are inconsistent, and the effect sizes of the associations are modest. Thus, the findings of the present review must be interpreted with caution. The lack of previous studies, the large heterogeneity among studies, inconsistent findings and modest effect sizes are, however, important information to be drawn from the present review.

The original research studies included in the present review often failed to correct for multiple comparisons and many did not consider the possible effects of confounders on the results. Therefore, some of the findings may be “false positive” findings and it is likely that confounders may have affected the results. Considering these key limitations of the original studies, the current study is valuable in the sense that it seeks to identify significant associations between psychological and immunological variables across studies using different psychological measures and assay systems to assess the same psychological construct and the same immunological parameter. In this context, it may be argued that a replicated finding indicates a true association.

Meta-analyses were not considered appropriate in the context of the present review due to the small number of eligible studies, the limitations of the original research studies and the heterogeneity among studies with regard to diagnostic criteria, psychological measures, and assay systems. Additional studies are needed that are both clinically and methodologically homogenous and more methodologically robust in order to conduct meaningful meta-analyses. Importantly, we believe that an attempt to further integrate the findings easily could have resulted in a form of overinterpretation of the findings. As meta-analyses were not conducted, possible limitations of the included studies did not contribute to any meta-analysis outcomes.

The present systematic review did not examine psychological and immunological differences between CFS/ME cases and healthy controls. Neither did this review consider which immunological variables (if any) are major in perpetuating a CFS/ME condition, or whether improvements in these specific markers of immunity in fact contribute to reduced symptom burden and recovery. Importantly, an association that is statistically significant is not necessarily biologically significant. A significant association may not be relevant to the biological processes of interest or useful from a practical point of view. Which psychological processes/health behaviors (if any) have noteworthy impact on the immune-related pathophysiology and manifestation of CFS/ME? This is an important aspect to consider in future research seeking to explore associations between psychological and immunological variables in CFS/ME.

## Conclusions

A growing body of evidence suggests that CFS/ME is associated with widespread dysfunction in the nervous, endocrine, and immune systems. Viewing CFS/ME through the lens of psychoneuroimmunology may thus contribute to uncover new perspectives on the vicious pathophysiological processes maintaining the illness and be an important next step toward the development of more effective treatments. On this basis, the present systematic review aimed to synthesize the available literature on (i) cross-sectional associations between psychological and immunological variables, and (ii) longitudinal associations between changes in psychological variables and changes in immunological variables, in CFS/ME. The review of the literature demonstrated that various psychological constructs are correlated with specific immunological parameters in CFS/ME. The results show that many key psychological constructs (executive function, emotion regulation, interpersonal function, sleep, and psychiatric symptoms) may be associated with cytokines, including IL-1β, IL-2(R), IL-6, and TNF-α. Poorer executive function, emotion regulation, interpersonal function, and sleep as well as higher levels of depressive symptoms and higher global psychiatric symptom intensity, have all been found to be associated with higher cytokine levels. Evidence further indicates that executive function and various symptom dimensions (mental health, psychiatric symptoms, and fatigue) might be associated with T cells (CD4^+^ and/or CD8^+^ counts/percentages). Only two associations, namely the association between sleep and IL-6 and the association between depression and IL-6, have been replicated twice. If the significant association between sleep and IL-6 was to be replicated in future studies, patients' quality of sleep could be investigated as a target of psychological intervention.

Although the present systematic review provides some novel insight into the nature of the associations between psychological and immunological variables in CFS/ME, conclusions cannot be drawn based on the limited amount of empirical evidence currently available. There are few consistent findings, and more research is needed to replicate and further explore the correlations between psychological and immunological variables in CFS/ME. In this regard, it is essential that researchers not only seek to examine psychoneuroimmunological relationships but also seek to develop and apply a more robust research methodology (e.g., by controlling for multiple comparisons). In order to facilitate comparison of results, it is further important that researchers seek to apply a more homogeneous research methodology across studies (e.g., by using similar psychological measures and assay systems). In addition, future studies should not only run correlational analyses but also make greater use of other statistical approaches (e.g., regression analyses) to shed light on possible psychoneuroimmunological relationships in CFS/ME. Furthermore, cause-and-effect relationships between variables cannot be inferred solely from the cross-sectional associations between them. Although nearly one-third of patients benefit substantially from psychological interventions, there is yet no evidence to suggest that changes in psychological variables during a psychological treatment for CFS/ME result in any similar changes in immunological parameters. Further, it is unknown whether improvements in immune system function contribute to reduced symptom burden and recovery from CFS/ME. More comprehensive, longitudinal studies are thus of vital importance to explore longitudinal associations between psychological and immunological changes, the associations between immunological and symptomatic changes as well as mediators and moderators of change. The results of such longitudinal studies will most likely provide important clues as to why some patients benefit from psychological treatments and some do not, and whether a psychological or pharmacological intervention is needed to successfully treat CFS/ME. Alternatively, various subtypes of CFS/ME may require different approaches to treatment. We are still missing pieces of the CFS/ME puzzle. Hopefully, this review will contribute to shed light on the intersection between mind and body and facilitate a refinement of current research efforts on CFS/ME.

## Data Availability Statement

The original contributions presented in the study are included in the article, further inquiries can be directed to the corresponding author.

## Author Contributions

ER and TS: conceptualization, design of the literature search, assessment of abstracts and/or full-text papers for inclusion, and writing—review and editing. ER: literature search, initial title and abstract screening, careful reading of other potentially relevant papers besides original research papers, screening of reference lists, writing—original draft preparation, and visualization. TS: supervision. All authors contributed to the article and approved the submitted version.

## Conflict of Interest

The authors declare that the research was conducted in the absence of any commercial or financial relationships that could be construed as a potential conflict of interest.

## Publisher's Note

All claims expressed in this article are solely those of the authors and do not necessarily represent those of their affiliated organizations, or those of the publisher, the editors and the reviewers. Any product that may be evaluated in this article, or claim that may be made by its manufacturer, is not guaranteed or endorsed by the publisher.
